# Berberine Chloride Suppresses Growth of Canine Osteosarcoma Cells via Regulating MMP2/AKT/β-Catenin Pathway

**DOI:** 10.3390/antiox15091094

**Published:** 2026-08-31

**Authors:** Minha Jeong, Juhyeong Seo, Sunwoo Park, Jiyeon Ham

**Affiliations:** 1 Department of Animal Science & Biotechnology, College of Agriculture and Life Sciences, Chungnam National University, Daejeon 34134, Republic of Korea; 202550625@o.cnu.ac.kr (M.J.); 202650483@o.cnu.ac.kr (J.S.); 2 Department of GreenBio Science, Gyeongsang National University, Jinju-si 52725, Republic of Korea

**Keywords:** osteosarcoma, viability, migration, ROS, calcium homeostasis

## Abstract

Canine osteosarcoma is an aggressive primary bone malignancy associated with a poor prognosis and a high propensity for lung metastasis. Due to the limitations and side effects of conventional chemotherapy, there remains a pressing need for alternative therapeutic strategies and adjuvants. In this study, we examined the effects of berberine chloride (BBRC), a bioactive isoquinoline alkaloid derived from the dried roots of *Coptidis Rhizoma*, on canine osteosarcoma D-17 and DSN cell lines. BBRC reduced the viability and migratory capacity of D-17 and DSN cell lines in three-dimensional (3D) spheroid cultures. Additionally, BBRC induced calcium ion overload in the mitochondrial matrix but not in the cytoplasm, in both osteosarcoma cell lines. BBRC also triggered excessive generation of reactive oxygen species (ROS) in both cell lines. Furthermore, Western Blot analysis revealed that BBRC downregulated the MMP2/AKT/β-catenin signaling pathways in both BBRC in D-17 and DSN cells. In addition, BBRC downregulated Cyclin D1 and Survivin, known downstream targets of the β-catenin pathway. We also confirmed that BBRC suppressed the transcription of genes involved in cell cycle regulation. Overall, our findings indicate that BBRC inhibits osteosarcoma cell growth by modulating calcium-redox homeostasis and the MMP2/AKT/β-catenin pathway.

## 1. Introduction

Canine osteosarcoma is the most prevalent primary malignant bone tumor. It arises from malignant transformation of immature bone-forming cells and is associated with a poor overall survival rate [1]. While osteosarcoma also primarily affects children and adolescents, it is also common in dogs, occurring approximately ten times more frequently in canines than in humans [1]. Canine osteosarcoma is characterized by aggressive clinical behavior, notably its high metastatic potential, with the lungs being the most common site of secondary spread [2]. Tumor localization in anatomical sites such as the proximal humerus, distal femur, and proximal tibia is associated with a higher risk of metastasis and, consequently, increased mortality [3,4]. The exact etiology of osteosarcoma in canine models remains poorly understood, where comprehensive molecular investigations are limited. Current standard treatment strategies for canine typically involve multimodal approaches combining surgical removal of tumor or adjuvant chemotherapy [5]. Although these regimens can prolong survival, their efficacy is limited by systemic toxicities, including nephrotoxicity from platinum-based compounds and cardiotoxicity from doxorubicin [6]. Moreover, conventional therapies are hindered by critical limitations, notably the development of chemoresistance in tumor cells and a severe decline in canine quality of life (QOL) due to treatment-associated toxicities [7]. Therefore, various efforts are underway to identify novel therapeutic candidates that can overcome the limitations of conventional chemotherapy, maximize the anti-tumor effects and suppression of metastasis in canine osteosarcoma, while minimizing toxicity to normal cells. In this context, oncology research has increasingly focused on compounds derived from natural products, which offer excellent biocompatibility and favorable safety profiles. Owing to their multi-target properties, natural products possess the potential to simultaneously modulate various oncogenic mechanisms—such as inhibiting cancer cell proliferation and suppressing both angiogenesis and metastasis—even as single agents. Furthermore, when administered in combination with conventional chemotherapeutics, they represent a highly promising strategy to overcome drug resistance through synergistic effects, thereby allowing for dose reduction and subsequent mitigation of adverse side effects in canine osteosarcoma models [8].

Among these natural compounds, berberine has emerged as a promising candidate. Berberine is an isoquinoline alkaloid extracted from the dried roots of *Coptidis Rhizoma*. It exhibits antibacterial, anti-inflammatory, antidiabetic, antiangiogenic, and cholesterol-lowering properties, as well as anticancer properties [9,10]. However, berberine exhibits extremely low oral bioavailability, estimated at approximately 0.68% [11]. To address this limitation, berberine chloride (BBRC), also known as berberine hydrochloride, was developed to enhance water solubility, absorption, and overall bioavailability [12]. BBRC, with its improved absorption characteristics, has demonstrated enhanced inhibitory effects on cancer cell migration and proliferation [13]. Specifically, berberine hydrochloride has demonstrated multiple biological activities, including anticancer and anti-inflammatory effects in organs such as the liver, lungs, and stomach [14,15,16]. Moreover, the chloride salt form could improve its aqueous solubility, thereby positively affecting its potential for higher bioavailability [12,17]. Additionally, a recent study demonstrated that berberine derivatives reduced cell viability and induced cell death in canine mammary gland tumor cells via inhibition of the Wnt/β-catenin signaling pathway [18]. These findings suggest that berberine derivatives are effective in canine cancer models, thereby expanding their potential applicability to a broader range of cancers, including osteosarcoma.

Therefore, in this study, we investigated the in vitro mechanism of action of BBRC in D-17 and DSN cell lines to explore its therapeutic potential as an adjunctive therapy for canine osteosarcoma, focusing on viability, migration, calcium and redox homeostasis, the MMP/AKT/β-catenin signaling pathway, and transcriptional regulation of cell cycle progression.

## 2. Materials and Methods

### 2.1. Cell Culture

Canine osteosarcoma cell lines D-17 (CVCL#1916, CCL-183) and DSN (CVCL#1919, CRL-9939), which were obtained from poodle, were purchased from the American Type Culture Collection (ATCC; Manassas, VA, USA) and cultured in Minimum Essential Medium (MEM; catalog no.: SH30024.01, HyClone, Logan, UT, USA) supplemented with 10% fetal bovine serum (FBS) at 37 °C in a humidified 5% CO_2_ incubator. Cells were cultured in 100 mm^2^ tissue culture dishes until reaching 70% confluency, serum-starved for 24 h, and subsequently treated with varying concentrations of BBRC. For assays, cells were seeded into 6-well plates at a density of 2 × 10^5^ cells/well and maintained until reaching 70% confluency. Cells were then serum-starved in serum-free medium for 24 h and treated with BBRC at concentrations of 0, 25, 50, and 75 μM for 48 h. Following treatment, both supernatants and adherent cells were collected by centrifugation for subsequent analysis.

### 2.2. Chemicals

BBRC (Berberine chloride; catalog no.: 00900585) and dimethyl sulfoxide (DMSO) were purchased from Sigma-Aldrich (St. Louis, MO, USA). N-acetyl-l-cysteine (NAC) (catalog no.: A7250) was purchased from Sigma-Aldrich. All compounds are > 95% pure by HPLC analysis.

### 2.3. MTT Assay for Measurement of Cell Viability

Cell viability was assessed using an MTT assay kit (catalog no. 11465007001; Roche, Indianapolis, IN, USA) following the manufacturer’s instructions. D-17 and DSN cells were seeded into 96-well plates and incubated in serum-free medium for 24 h. Cells were then treated with BBRC at concentrations of 0, 25, 50, or 75 µM and incubated for either 48 or 72 h. After incubation, 10 μL of MTT reagent was added to each well under light-protected conditions. The cells were further incubated for 4 h to allow the formation of purple formazan crystals. After crystal formation, 100 μL of detergent reagent was added to each well. The plates were incubated overnight, and absorbance was measured at 570 nm the next day using an ELISA reader.

### 2.4. Spheroid Culture

D-17 and DSN cells were suspended at a concentration of 1 × 10^6^ cells/mL. Treatment solutions were prepared by adding either DMSO alone (vehicle control) or BBRC (75 μM) to the cell suspension. A 25 μL aliquot (6000 cells/drop) of each suspension was carefully dispensed vertically onto the inner surface of a sterile 100 mm^2^ Petri dish lid. Phosphate-buffered saline (PBS) was added to the bottom of the dish to prevent evaporation. The cells were incubated at 37 °C in a humidified 5% CO_2_ atmosphere for five days to allow cell aggregation and spheroid formation. Spheroid formation in the vehicle and BBRC (75 μM) treatment groups was monitored daily at the same time using an EVOS M5000 imaging system.

### 2.5. Wound-Healing Assay

The migratory capacity of D-17 and DSN cells was assessed using an ibidi culture-insert 2-well dish (catalog no.: 81176). Cells were seeded into each well at a density of 1 × 10^6^ cells/mL and incubated until confluence. After the insert chambers were removed, media containing either vehicle control or BBRC (75 μM) were added. Images were captured at 0, 6, and 24 h using an EVOS M5000 imaging system (Thermo Fisher Scientific, Waltham, MA, USA). For quantitative analysis, the wound closure area between cell monolayers was measured using ImageJ software (version 1.54f).

### 2.6. Analysis of Relative Calcium Ion Levels in the Cytosol and Mitochondrial Matrix

To measure cytosolic calcium levels, 3 μM Fluo-4 AM (Invitrogen, Carlsbad, CA, USA) was prepared and added to the cells at 500 μL per well. The cells were incubated for 20 min at 37 °C in a 5% CO_2_ humidified incubator. After staining, the cells were washed twice with PBS and prepared for flow cytometry analysis. Mitochondrial calcium levels were assessed using Rhod-2 AM (3 μM) diluted in Hank’s Balanced Salt Solution (HBSS; Gibco) and added to the cells. Cells were incubated at 4 °C for 30 min in the dark. After staining, Rhod-2 AM-labeled cells were washed with HBSS and incubated again at 37 °C for 10 min. Finally, cells were resuspended in buffer and analyzed via flow cytometry.

### 2.7. Western Blot Analysis

Protein concentrations were determined using the Bradford assay, with bovine serum albumin (BSA) as the standard. Proteins (25 μg/well) were denatured, separated by sodium dodecyl sulfate-polyacrylamide gel electrophoresis (SDS-PAGE), and transferred onto nitrocellulose membranes. Protein bands were visualized using an enhanced chemiluminescence detection system (SuperSignal West Pico), and band intensities were quantified under ultraviolet illumination using an Amersham ImageQuant 800 system (Cytiva). Primary antibodies used in this study are listed in Table 1. Goat anti-rabbit and goat anti-mouse polyclonal antibodies were employed as secondary antibodies. Each immunoblot was normalized to total protein or β-actin as a loading control. All antibodies were purchased from Cell Signaling Technology.

### 2.8. Determination of Cellular Reactive Oxygen Species (ROS)

Intracellular reactive oxygen species (ROS) levels were measured by the conversion of 2′,7′-dichlorofluorescin diacetate (DCFH-DA; Sigma-Aldrich) into the fluorescent compound 2′,7′-dichlorofluorescin (DCF). Adherent cells were detached using trypsin-EDTA, collected by centrifugation, and washed with PBS. Cells were incubated with 10 µM DCFH-DA at 37 °C for 30 min in the dark. After incubation, cells were washed twice with PBS, and DCF fluorescence intensity was analyzed using a CytoFLEX flow cytometer (Beckman Coulter, Brea, CA, USA).

### 2.9. Immunofluorescence for β-Catenin

To assess the nuclear translocation of β-catenin, immunofluorescence microscopy was performed in DSN and D-17 cells. Both cells were seeded at confocal dish as concentration of 1.5 × 10^4^ cells per dish. Then, cells were treated for 1 h with or without NAC (2 mM), followed by BBRC (75 μM) treatment for 24 h. Cells were probed with an anti-β-catenin antibody (catalog no. 37447, Cell Signaling Technology, Danvers, MA, USA) at a final dilution of 1:4200 and a goat anti-mouse IgG Alexa 594 (catalog no. A-11005, Invitrogen, Carlsbad, CA, USA) at 6 μg/mL at room temperature for 1 h. Then, both cell lines were washed with 0.1% BSA in PBS and overlaid with 4′,6-diamidino-2-phenylindole (DAPI). Images were captured using an LSM-880 Carl Zeiss microscopy GmbH (Oberkochen, Germany).

### 2.10. Quantitative Real-Time Polymerase Chain Reaction (PCR) Analysis

To assess target gene expression at the mRNA level, total RNA was extracted from D-17 and DSN cells treated with either DMSO (vehicle control) or 75 μM BBRC for 24 h using TRIzol reagent (catalog no. 1023537). Complementary DNA (cDNA) was synthesized from the extracted RNA using a SimpliAmp thermal cycler (Applied Biosystems, Singapore). In detail, extracted RNA (1 μg) with random primer and oligo dT were incubated at 70 °C for 5 min. And then AccuPower^®^ RT PreMix (Bioneer, catalog no. K-2041) was added. After that, the mixtures were incubated at 42 °C for 60 min, and 94 °C for 5 min in thermal cycler. Quantitative real-time PCR (qRT-PCR) was conducted using the CFX Duet system. Amplification was carried out with an initial denaturation at 95 °C for 3 min, followed by 40 cycles of 95 °C for 30 s, 60 °C for 30 s, and 72 °C for 30 s. The Ct values obtained from the technical triplicates were averaged and used to calculate relative mRNA expression using the 2^−ΔΔCt^ method. The primers used in this study are listed in Table 2. *GAPDH* was used as the internal reference gene for normalization.

### 2.11. Statistical Analysis

Data from proliferation assays and Western Blot analyses were analyzed by one-way analysis of variance (ANOVA) using the general linear model (PROC-GLM) in SAS 9.4 software (SAS Institute, Cary, NC, USA) to evaluate the effects of treatment on signal transduction pathways in D-17 and DSN cells. All experiments were performed in triplicate, and the resulting data were used for statistical analysis. Differences were considered statistically significant at *p* < 0.05. Data are shown as the mean ± SD of three independent biological replicates unless otherwise specified.

## 3. Results

### 3.1. Berberine Chloride (BBRC) Suppresses the Viability of Canine Osteosarcoma D-17 and DSN Cells

**Figure 1:**
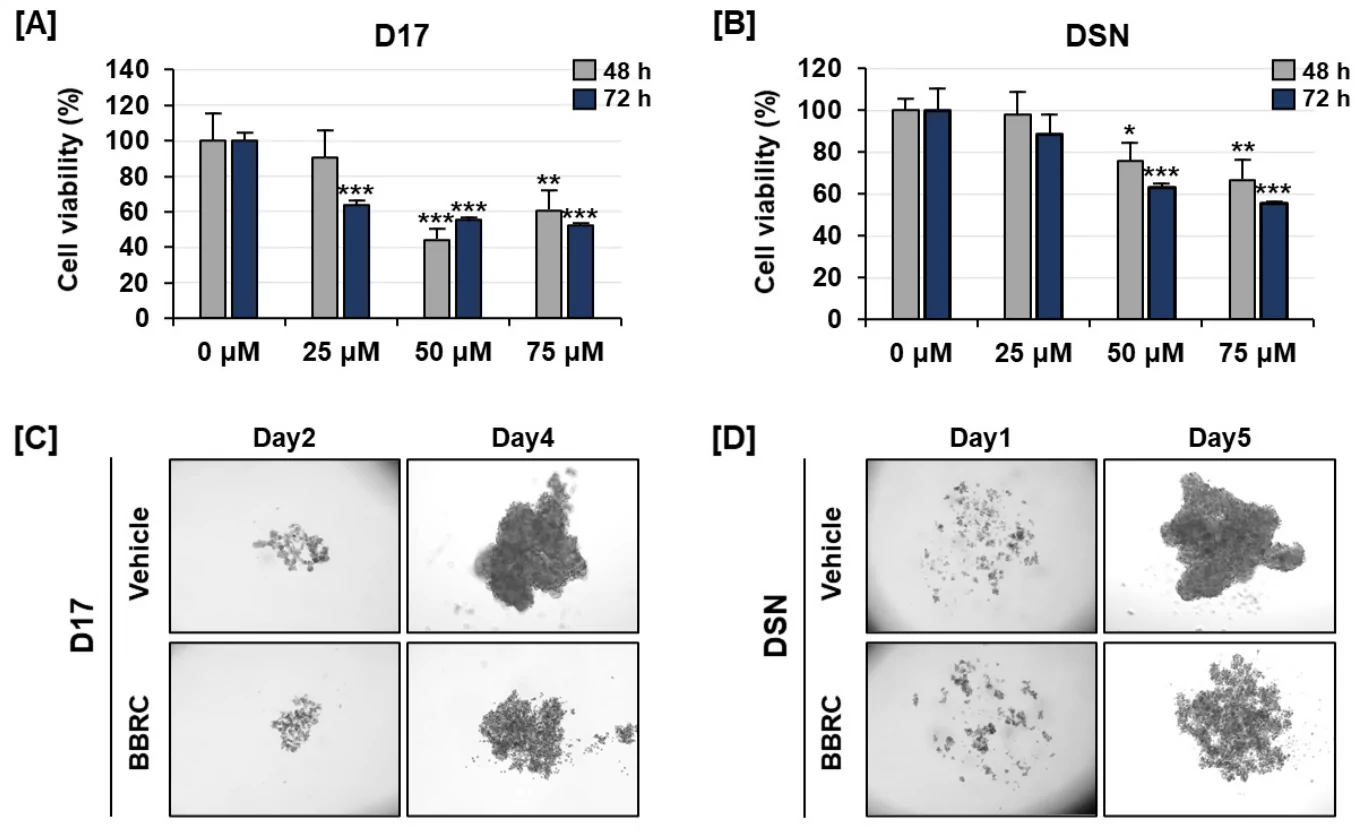
Suppressive effects of BBRC on canine osteosarcoma cell lines. (**A**,**B**) Cell viability was measured D-17 and DSN cells using the MTT assay after treatment with BBRC (0, 25, 50, and 75 μM) for 48 and 72 h. (**C**,**D**) In 3D spheroid cultures, BBRC (75 μM) inhibited spheroid formation in both cell lines, indicating reduced proliferative activity. Asterisks indicate statistically significant treatment effects (* *p * <  0.05, ** *p*  <  0.01, and *** *p*  <  0.001).

To investigate the therapeutic effects of BBRC on canine osteosarcoma cells (D-17 and DSN), cells were treated with increasing concentrations of BBRC (0, 25, 50, and 75 μM) for 48 and 72 h. Treatment with 75 μM BBRC significantly reduced the viability of D-17 cells to 61% and 52% at 48 and 72 h, respectively (Figure 1A). Similarly, in DSN cells, viability gradually decreased, reaching approximately 67% at 48 h and 56% at 72 h following 75 μM BBRC treatment (Figure 1B). To further validate the antiproliferative effects of BBRC, we employed three-dimensional (3D) spheroid cultures of D-17 and DSN cells. Cells were treated with 75 μM BBRC and cultured for 4 to 5 days, with images captured every 24 h for analysis (Figure 1C,D). After 4–5 days of incubation, vehicle-treated cells formed dense, compact spheroids, whereas BBRC-treated D-17 and DSN cells failed to aggregate and remained dispersed (Figure 1C,D). Collectively, these results indicate that BBRC inhibits the proliferation of canine osteosarcoma cells.

### 3.2. BBRC Inhibits Migration in D-17 and DSN Cells

**Figure 2:**
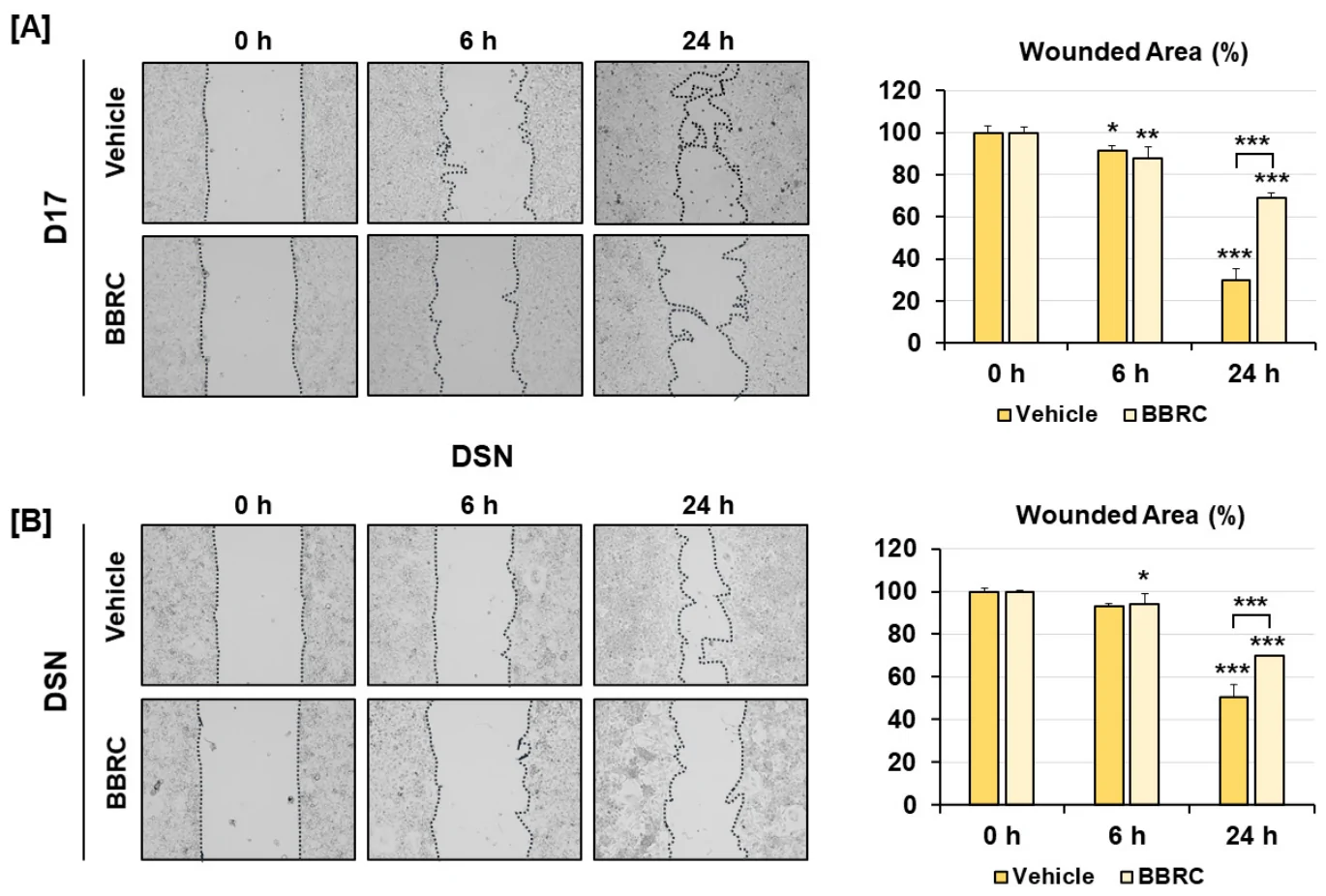
Inhibition of canine osteosarcoma cell migration by BBRC. (**A**,**B**) Wound-healing assays were conducted to assess the migratory capacity of D-17 and DSN cells treated with BBRC (75 μM) for 24 h. Compared with the vehicle group, BBRC-treated cells exhibited delayed wound closure in both cell lines. Wound areas were quantified using ImageJ software (version 1.54f). Comparisons were made between vehicle groups over time, BBRC-treated groups over time, and between vehicle and treated groups at the same time points (* *p*  <  0.05, ** *p*  <  0.01, and *** *p*  <  0.001).

To assess cell migration, D-17 and DSN cells were treated with BBRC (75 μM) for 24 h, and wounds were analyzed. At 6 h post-treatment, both vehicle- and BBRC-treated cells showed no significant difference in wound closure in either cell lines (Figure 2A,B). However, after 24 h, BBRC-treated cells in both lines exhibited delayed wound closure compared to vehicle-treated cells. In D-17 cells, approximately 70% of the wound area was closed in the vehicle group, whereas only 31% closure was observed in the BBRC-treated group (Figure 2A). Similarly, in DSN cells, the vehicle group showed 50% wound closure after 24 h, whereas the BBRC-treated group exhibited only 30% wound closure (Figure 2B).

### 3.3. BBRC Disrupts Calcium Homeostasis in D-17 and DSN Cells

**Figure 3:**
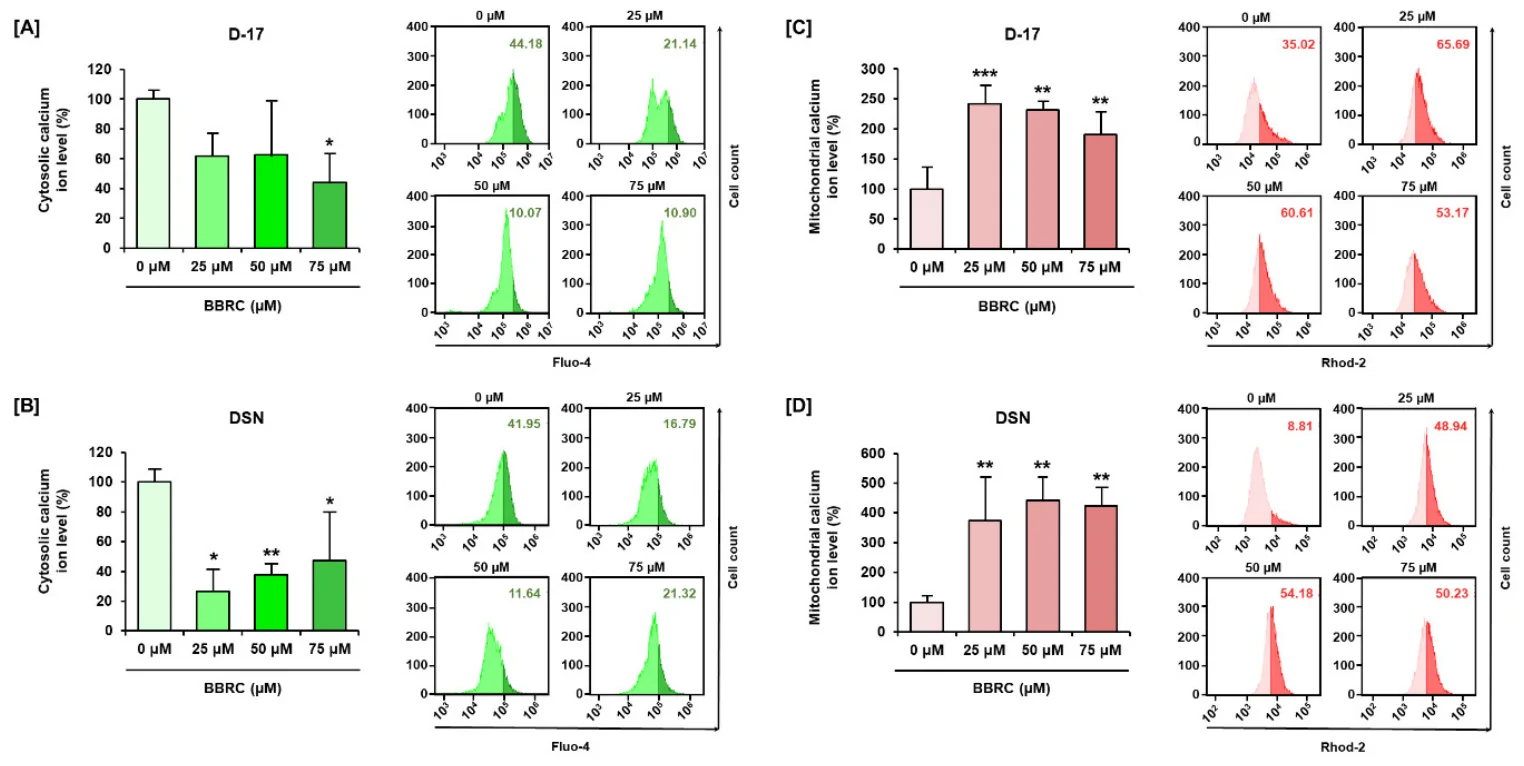
Disruption of calcium homeostasis by BBRC in canine osteosarcoma cells. (**A**,**B**) Cytosolic calcium levels in D-17 and DSN cells were assessed using Fluo-4 AM staining following BBRC treatment (0, 25, 50, 75 μM). BBRC significantly reduced cytosolic calcium levels in both cell lines. (**C**,**D**) Mitochondrial calcium levels were measured using Rhod-2 AM staining. BBRC treatment led to a dose-dependent increase in mitochondrial calcium accumulation in D-17 and DSN cells. Asterisks indicate statistically significant treatment effects (* *p*  <  0.05, ** *p*  <  0.01, and *** *p*  <  0.001).

We next examined calcium accumulation in the cytosol and the mitochondrial matrix of D-17 and DSN cells. In D-17 cells, cytosolic calcium levels decreased by approximately 44%, while DSN cells exhibited a sharp reduction to about 47% following 75 µM BBRC treatment (Figure 3A,B). In D-17 cells, mitochondrial calcium levels increased more than 1.9-fold following dose-dependent BBRC treatment (Figure 3C). In DSN cells, mitochondrial calcium levels increased by 4.4- and 4.2-fold following 50 and 75 µM BBRC treatments, respectively (Figure 3D). Collectively, BBRC caused a marked reduction in cytosolic calcium levels and an abnormal increase in mitochondrial matrix calcium levels in osteosarcoma cells. These findings suggest that BBRC-induced disruption of calcium homeostasis may contribute to mitochondrial dysfunction.

### 3.4. BBRC Regulates MMP2/AKT/β-Catenin Pathway in Canine Osteosarcoma Cells

**Figure 4:**
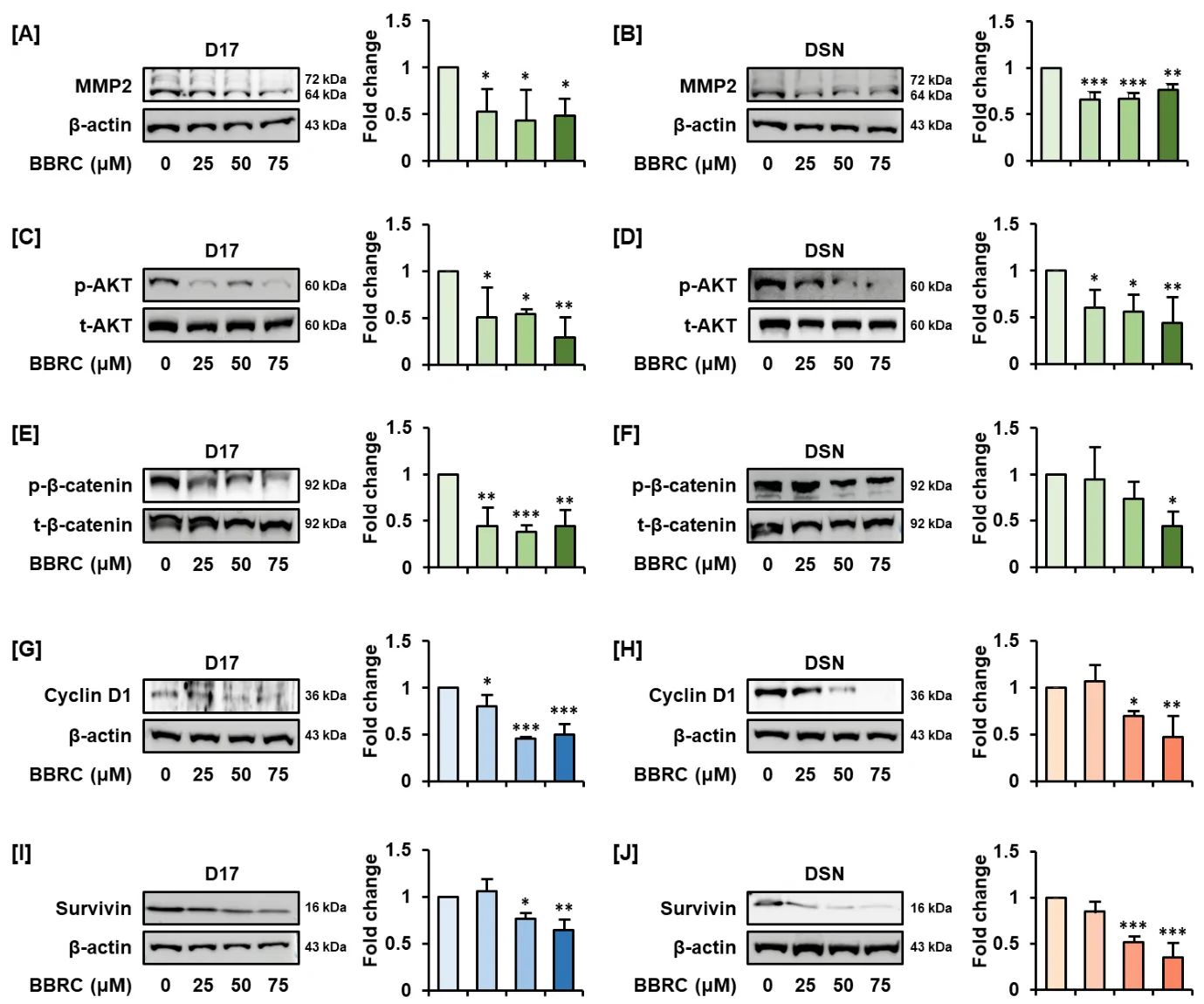
Suppression of the MMP2/AKT/β-catenin signaling pathway by BBRC in canine osteosarcoma cells. Western Blot analysis of MMP2, phosphorylated AKT (p-AKT, Ser473), β-catenin, Cyclin D1 and Survivin in D-17 and DSN cells treated with BBRC (0, 25, 50, and 75 μM) for 24 h. (**A**,**B**) MMP2 levels in D-17 and DSN cells. (**C**,**D**) Phosphorylated AKT (p-AKT) levels in D-17 and DSN cells. (**E**,**F**) Phospho-β-catenin (Ser552) levels in D-17 and DSN cells. (**G**,**H**) Cyclin D1 levels in D-17 and DSN cells. (**I**,**J**) Survivin levels in D-17 and DSN cells. Total proteins and β-actin were used as loading controls. Asterisks indicate statistically significant effects of treatment (* *p*  <  0.05, ** *p*  <  0.01, and *** *p*  <  0.001).

To investigate intracellular signaling pathways affected by BBRC in osteosarcoma cells, we performed Western Blot analysis focusing on the MMP2/AKT/β-catenin pathways, which are associated with cell viability and cell cycle progression (Figure 4). BBRC treatment (0, 25, 50, and 75 μM) reduced MMP2 expression to 0.49-fold compared to the untreated control in D-17 cells (Figure 4A). Similarly, MMP2 expression was reduced to 0.77-fold in DSN cells following BBRC treatment (Figure 4B). Phosphorylation of AKT was also significantly inhibited, decreasing to less than 0.5-fold in both D-17 and DSN cells following BBRC treatment (Figure 4C,D). β-catenin phosphorylation levels were suppressed in a dose-dependent manner in both cell lines (Figure 4E,F). In addition, expression of Cyclin D1 and Survivin significantly decreased in a dose-dependent manner in both BBRC-treated D-17 and DSN cells (Figure 4G–J). Collectively, these findings indicate that BBRC suppresses the MMP2/AKT/β-catenin signaling pathway in canine osteosarcoma cells.

### 3.5. BBRC Generates Excessive Oxidative Stress in Osteosarcoma Cells

**Figure 5:**
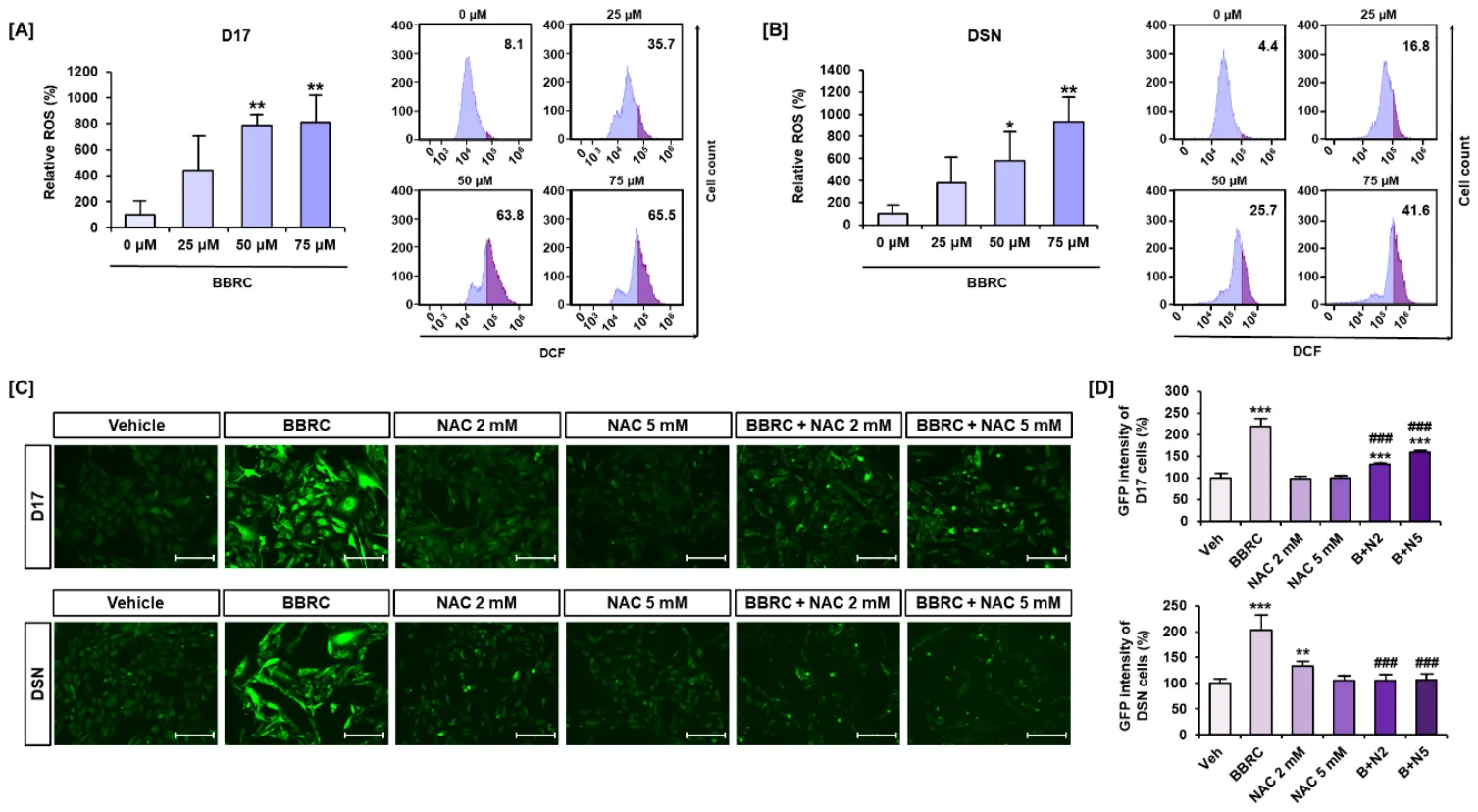
BBRC induces oxidative stress in canine osteosarcoma cells. (**A**,**B**) Intracellular ROS levels were measured by flow cytometry in suspension cultures of D-17 and DSN cells treated with BBRC (0, 25, 50, and 75 μM) for 1 h. ROS-positive cells were detected using DCFH-DA staining. (**C**) DCF, the oxidized product of ROS, was visualized as green fluorescence. Images were captured using an EVOS M5000 system. (**D**) Relative fluorescence intensities were quantified using the software built into the EVOS M5000. Scale bar: 150 μm. Asterisks indicate statistical significance between vehicle and treated groups (* *p*  <  0.05, ** *p*  <  0.01, and *** *p*  <  0.001). Crosshatches indicate the significance between BBRC and BBRC with NAC co-treatment group (### *p* < 0.001).

To evaluate whether BBRC treatment induces oxidative stress, we measured intracellular ROS levels. In D-17 cells, ROS levels increased in a dose-dependent manner, reaching an 8.1-fold elevation at 75 μM BBRC treatment (Figure 5A). In DSN cells, BBRC treatment led to a 9.3-fold increase in intracellular ROS levels (Figure 5B). To confirm the role of oxidative stress in BBRC-mediated effects, cells were co-treated with BBRC and the ROS scavenger N-acetylcysteine (NAC). ROS levels were visualized by measuring green fluorescence intensity using an EVOS microscope (Figure 5C). Consistent with flow cytometry results, BBRC (75 μM)-treated cells exhibited more than a two-fold increase in green fluorescence compared to vehicle-treated cells in both D-17 and DSN cells (Figure 5D). NAC treatment (2 or 5 mM) did not induce substantial changes, except in the 2 mM NAC-treated DSN cells. In D-17 cells, NAC co-treatment (2 and 5 mM) reduced BBRC-induced ROS levels to 131% and 159%, respectively. Additionally, in DSN cells, NAC co-treatment restored BBRC-induced ROS levels to near-baseline levels (Figure 5D).

### 3.6. BBRC Induces Mild Cytosolic Redistribution of Total-β-Catenin in Canine Osteosarcoma Cells

**Figure 6:**
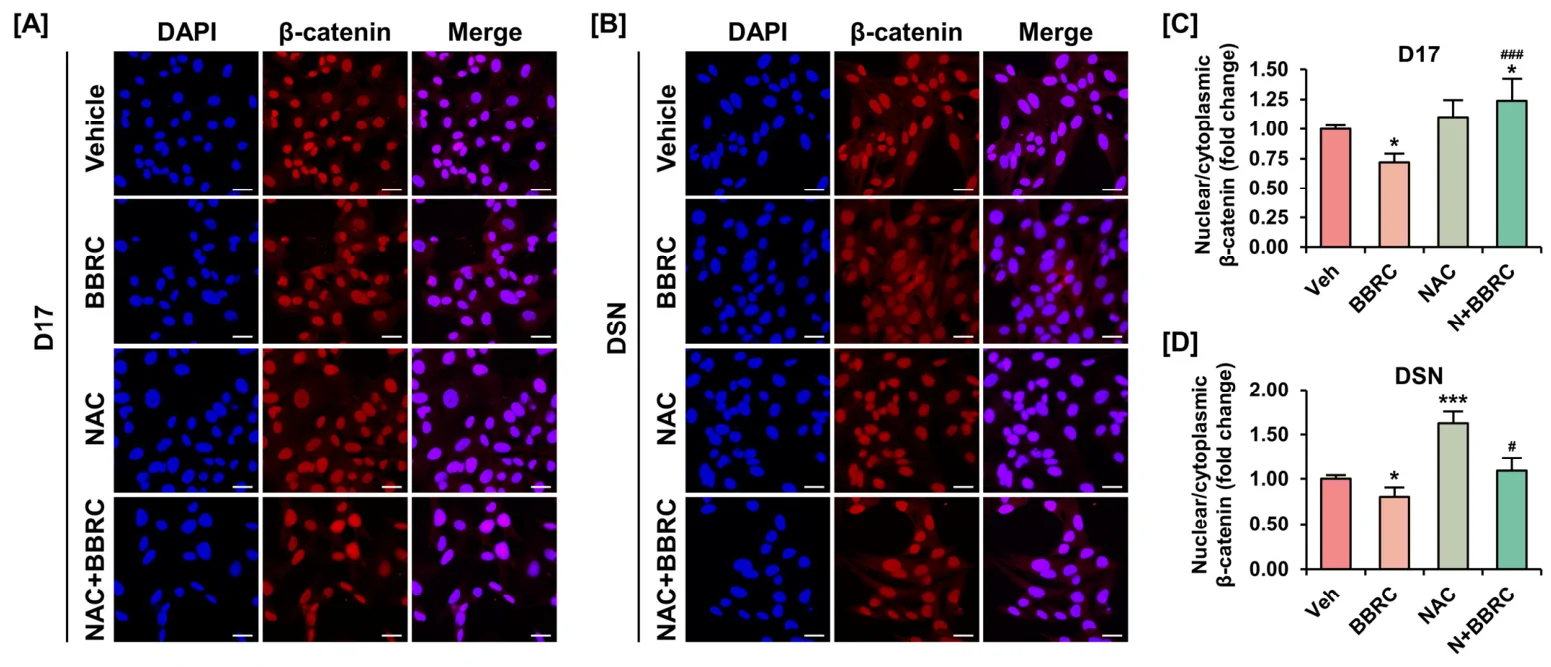
BBRC partially restricts the nuclear translocation of β-catenin in D-17 and DSN cells. (**A**,**B**) Immunofluorescence images indicate the subcellular localization of β-catenin in the nucleus and cytosol of canine osteosarcoma cells. Both cells were treated with BBRC (75 μM) for 24 h with or without pre-treatment of NAC (2 mM) for 1 h. Nuclei were counterstained with DAPI (blue) for co-localization, and β-catenin were labeled with Alexa594 (Red). Scale bar = 30 μm. (**C**,**D**) Relative nuclear/cytoplasmic β-catenin ratio (red fluorescence intensity) was analyzed using Image J software. Asterisks indicate statistical significance between vehicle and treated groups (* *p*  <  0.05, and *** *p*  <  0.001). Crosshatches indicate the significance between BBRC and BBRC with NAC co-treatment group (# *p*  <  0.05, and ### *p*  <  0.001).

As a transcription factor, β-catenin can translocate to the nucleus upon phosphorylation at Ser552. Therefore, we further investigated the effects of BBRC on the subcellular localization of β-catenin using an immunofluorescence (IF) assay. Furthermore, to determine whether this effect is mediated by BBRC-induced oxidative stress, cells were pre-treated with NAC (2 mM). In the vehicle group, β-catenin was predominately localized in the nucleus; however, upon exposure to BBRC, a mild cytosolic redistribution of β-catenin was observed in both D-17 and DSN cells, although a considerable amount still remained in the nucleus (Figure 6A,B). Notably, pre-treatment with NAC effectively maintained the strong nuclear accumulation of β-catenin, counteracting the BBRC-induced changes (Figure 6A,B). In D-17 cells, the nuclear/cytoplasmic β-catenin ratio decreased significantly to 0.72-fold compared to the control group following BBRC treatment and recovered to 1.24-fold with NAC pre-treatment (Figure 6C). Similarly, in DSN cells, the nuclear/cytoplasmic ratio decreased to approximately 0.8-fold upon exposure to BBRC but recovered significantly to its basic level with NAC pre-treatment (Figure 6D). Taken together, these findings suggest that BBRC partially restricts the nuclear translocation of β-catenin through an ROS-dependent mechanism.

### 3.7. BBRC Regulates Cell Cycle Progression at the Transcriptional Level in Osteosarcoma Cells

**Figure 7:**
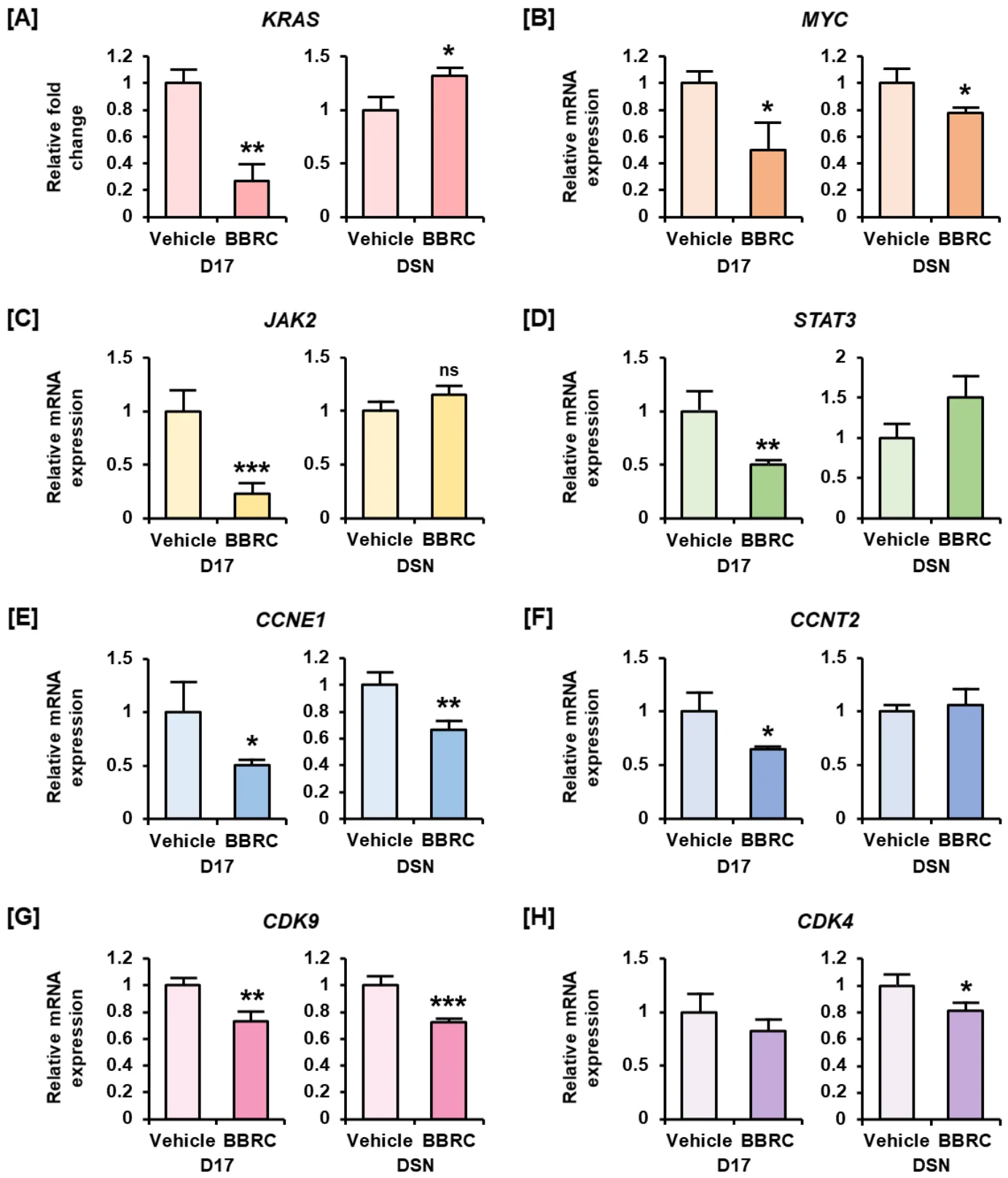
Modulation of proliferation and cell cycle-related gene expression by BBRC. (**A**–**H**) qRT-PCR analysis of mRNA expression in D-17 and DSN cells treated with BBRC (75 μM) for 24 h. Genes analyzed include *KRAS*, *MYC*, *JAK2*, *STAT3*, *CCNE1*, *CCNT2*, *CDK9*, and *CDK4*. Asterisks indicate statistically significant treatment effects (* *p*  <  0.05, ** *p*  <  0.01, and *** *p*  <  0.001). ‘ns’ indicates non-significant differences.

qRT-PCR was performed to evaluate the mRNA expression of genes related to tumor growth, metastasis, and cell cycle progression in canine osteosarcoma cells. Treatment with 75 μM BBRC reduced *KRAS* mRNA expression to 27% in D-17 cells, whereas expression increased 1.3-fold in DSN cells (Figure 7A). In addition, *MYC* expression decreased by approximately 50% in D-17 cells and 78% in DSN cells (Figure 7B). *JAK2* and *STAT3* mRNA expression significantly decreased in D-17 cells, but not in DSN cells (Figure 7C,D). *CCNE1* expression decreased by approximately 51% in D-17 cells and 67% in DSN cells (Figure 7E). *CCNT2* expression decreased in D-17 cells, whereas no significant change was detected in DSN cells (Figure 7F). *CDK9*, a member of the cyclin-dependent kinase (CDKs) family, showed a 72–73% reduction in mRNA expression in both BBRC-treated D-17 and DSN cells (Figure 7G). *CDK4* mRNA levels were also reduced by approximately 20% following BBRC treatment, with statistical significance observed only in DSN cells (Figure 7H). Collectively, these findings demonstrate that BBRC modulates the transcription of genes involved in cell proliferation and cell cycle regulation in osteosarcoma cells, thereby inhibiting tumorigenesis.

## 4. Discussion

In this study, berberine chloride significantly reduced cell viability and suppressed the migration of canine osteosarcoma cell lines D-17 and DSN. In addition, it increased intracellular ROS levels, reduced cytosolic calcium concentrations, and inactivated the MMP2/AKT/β-catenin signaling pathway, leading to downregulation of Cyclin D1 and Survivin expression (Figure 7). These findings suggest that BBRC inhibits cell proliferation of canine osteosarcoma cells by promoting mitochondrial dysfunction, enhancing oxidative stress, and suppressing key survival signaling pathways.

Osteosarcoma is the most common primary malignant bone tumor in both humans and dogs, accounting for approximately 85% of all skeletal tumors in canines [19]. It consists of malignant mesenchymal cells and is characterized by highly aggressive biological behavior and significant metastatic potential [2]. And berberine has been reported to induce cytotoxicity, DNA damage, and nuclear fragmentation in the human osteosarcoma cell line MG-63, suggesting that it could be a promising candidate for suppressing cancer growth and metastasis [20]. Previous studies have demonstrated that berberine hydrochloride exerts anti-proliferative and anti-metastatic effects in various cancer models, such as non-small cell lung cancer, nasopharyngeal carcinoma, and gallbladder cancer [15,21,22]. Although several studies have demonstrated the anticancer effects of berberine in human models, its therapeutic efficacy in dogs remains inadequately established. Despite significant genetic homology between humans and dogs, species-specific differences in cellular responses may influence drug efficacy [23]. Given this research gap, we investigated the anticancer mechanisms of BBRC in canine osteosarcoma cell lines.

Mitochondria are essential organelles that regulate various cellular metabolic functions, including ATP production, calcium homeostasis, cytotoxicity, and redox homeostasis [24,25,26]. In our study, mitochondrial calcium uptake was observed and was presumed to originate primarily from cytosolic calcium [27]. The endoplasmic reticulum (ER) and mitochondria are the principal calcium reservoirs, and excessive calcium accumulation in the ER can trigger ER stress [28]. However, our results showed decreased cytoplasmic calcium ion levels, whereas an increase in mitochondrial matrix (Figure 3). Plus, activation of the ER stress was not detected by BBRC. Therefore, mitochondrial calcium sequestration is likely to involve the uptake of calcium ions from the cytosol, which is triggered by external stimuli such as BBRC [29]. Mitochondrial calcium accumulation can lead to cytochrome c release and activation of the intrinsic apoptotic pathway, resulting in programmed cell death [30,31]. Consistent with these findings, we observed a decrease in the cytosolic calcium levels and a corresponding increase in mitochondrial calcium levels using flow cytometry. These results suggest that BBRC induces calcium influx from the cytosol into the mitochondria, thereby disrupting calcium homeostasis in canine osteosarcoma cells.

In parallel with mitochondrial dysfunction, we also observed dysregulated redox homeostasis, a major function of mitochondria [32]. Cancer cells typically exhibit higher basal levels of ROS than normal cells. However, excessive ROS generation following BBRC treatment can trigger cell death even in cancer cells [33]. ROS overproduction can lead to mitochondrial dysfunction and inhibition of cell proliferation [34,35]. Furthermore, co-treatment with NAC, a ROS scavenger, reversed BBRC-induced ROS accumulation. These findings suggest that BBRC exerts its antiproliferative and antimetastatic effects on D-17 and DSN cells in a ROS-dependent manner.

In this study, we confirmed that berberine chloride modulates the MMP/AKT/β-catenin signaling pathway. AKT promotes several cellular metabolic processes, including cell survival, proliferation, and metabolic regulation [36]. In the present study, BBRC inhibited AKT pathway activity, thereby suppressing the survival and proliferation of canine osteosarcoma cells. Activated AKT also phosphorylates β-catenin at the Ser552 residue [37]. Phosphorylation at this site regulates the transcriptional activity of β-catenin, which functions as a transcription factor that translocate to the nucleus and promotes gene expression [38]. This transcriptional activity induces epithelial-to-mesenchymal transition (EMT) and upregulates expression of metastasis-associated proteins such as MMP-2 and MMP-9. MMP-2 and MMP-9 are metastasis-promoting enzymes that degrade the extracellular matrix (ECM), facilitating cancer cell invasion and migration [39,40,41]. In our study, BBRC mildly suppressed nuclear translocation of β-catenin and MMP2 protein expression as well as cell migration capacity. In addition to regulating migration, β-catenin activates Cyclin D1 and Survivin. Cyclin D1 promotes the G1-to-S phase transition by phosphorylating the retinoblastoma protein (Rb), thereby facilitating cell cycle progression [42]. Survivin is an anti-apoptotic protein that regulates the cell cycle progression and promotes cell survival. BBRC inhibits β-catenin, thereby suppressing Cyclin D1 and Survivin expression, which in turn disrupts cell cycle progression and cell survival, consistent with our results in Figure 1 and Figure 6. Thus, BBRC modulates the MMP/AKT/β-catenin pathway, thereby inducing cell cycle arrest and subsequent inhibition of cell growth, proliferation, and metastasis.

To further clarify the mechanism of cell cycle arrest, we analyzed changes in mRNA expression of genes related to the cell cycle and proliferation using qPCR. Frequent mutations in the MAPK/PI3K signaling cascade, including the prominent oncogene *KRAS* responsible for promoting tumor growth and proliferation, have been identified via whole-genome sequencing as a major pathogenic mechanism of canine osteosarcoma [43,44]. The Wnt/β-catenin signaling pathway is also present in canine osteosarcoma, and similar oncogenic expression patterns of KRAS and β-catenin have been observed in both human and canine breast cancers [45,46]. *MYC* is a transcription factor that plays a key role in tumor initiation and sustained growth by regulating the cell cycle and promoting metastasis [47]. In particular, *Myc* is widely known to be frequently overexpressed in human osteosarcoma, and it is a representative target gene whose expression is induced when β-catenin translocate to the nucleus and binds to TCF/LEF. This finding is consistent with our immunofluorescence analysis of β-catenin and our qPCR data. The JAK2/STAT3 pathway promotes cell proliferation and differentiation in both canines and humans [48,49]. Cyclin E1, encoded by *CCNE1*, forms a complex with CDK2 that phosphorylates Rb and plays a central role in regulating the G1-to-S phase transition. Overexpression of *CCNE1* shortens the G1 phase and accelerates entry into the S phase, thereby increasing tumorigenic potential [50]. A genome-wide association study (GWAS) of canine osteosarcoma has also demonstrated that mutations in these cell cycle regulators are involved in the onset of the disease [51]. Cyclin T2, encoded by *CCNT2*, forms a complex with CDK9 known as Positive Transcription Elongation Factor B (P-TEFb), which regulates transcriptional elongation and contributes to cell proliferation [52]. CDK9 is also overexpressed in human osteosarcoma cells, and its inhibition reduces cell proliferation and induces apoptosis [53]. CDK4, another member of the cyclin-dependent kinase (CDK) family, forms a complex with Cyclin D1 to regulate the cell cycle and promote progression, growth, and proliferation in osteosarcoma cells [54,55]. It has been reported that such CDK4 overexpression, abnormal regulation of Rb, and abnormalities in the G1/S and G2/M checkpoints occur in both canine and human [56]. The differential transcriptional responses of upstream signaling molecules, notably *KRAS* and *JAK2/STAT3*, between D-17 and DSN cells can be attributed to their distinct genetic backgrounds. D-17 originates from metastatic osteosarcoma, whereas DSN is a genetically modified derivative of D-17, serving as a helper cell line designed to express viral core genes (*gag*, *pol*, *env*, and *Tn5 Neo*) [57]. The stable expression of these retroviral genes may indirectly affect cellular susceptibility to cytotoxic stress and transcriptional regulation. Importantly, despite these disparate upstream responses, the expression of master downstream effectors—*MYC*, *CCNE1*, and *CDK9* —was consistently and significantly suppressed in both cell lines. This suggests that BBRC can effectively block common downstream execution steps, ultimately inhibiting cell viability and metastasis, regardless of the complex upstream mutations present in individual cell lines.

Although most research on berberine’s anticancer effects has focused on human cancers, this study is notable as one of the few investigating its mechanism of action in canine osteosarcoma cells. Given the similarities in tumor biology between dogs and humans, these findings highlight BBRC’s potential as a therapeutic agent in veterinary oncology. Therefore, BBRC may serve as a promising natural compound as an adjunctive therapy to complement conventional osteosarcoma treatments.

A primary limitation of our study is that it was confined to in vitro experiments. Additionally, this research relies on D-17 and its derivative, DSN, which share a common genetic origin (Poodle). Given the profound intra- and inter-tumoral heterogeneity of canine osteosarcoma across different breeds, the generalizability of BBRC’s efficacy must be further validated. Without preclinical animal models, we could not directly confirm whether BBRC exhibits lower in vivo toxicity compared to conventional anticancer drugs, nor could we assess its potential metabolic toxicity. Furthermore, we were unable to provide definitive evidence demonstrating that BBRC has higher bioavailability than berberine. Therefore, further preclinical studies are essential to verify the in vivo efficacy of BBRC and to investigate its use in combination therapies.

## 5. Conclusions

In conclusion, this study demonstrated that berberine exhibits significant anticancer activity against canine osteosarcoma cells (D-17 and DSN). Berberine inhibited cell migration and proliferation, increased intracellular ROS production, and reduced cytosolic calcium levels, leading to the loss of mitochondrial membrane potential. Furthermore, it inactivated the MMP/AKT/β-catenin signaling pathway, resulting in the downregulation of Cyclin D1 and Survivin, which are mainly involved in cell survival and proliferation. 

## Figures and Tables

**Table 1:** List of antibodies used for Western Blot.

Primary Antibodies	Supplier	Catalog Number	Species	Dilution	RRID Number
MMP2	Cell Signaling Technology	4022	Rabbit	1:1000	AB_2266622
Phospho-AKT (Ser473)	Cell Signaling Technology	4060	Rabbit	1:1000	AB_2315049
Total AKT	Cell Signaling Technology	9272	Rabbit	1:1000	AB_329827
Phospho-β-catenin (Ser552)	Cell Signaling Technology	9566	Rabbit	1:1000	AB_1031116
Total β-catenin	Cell Signaling Technology	37447	Mouse	1:1000	AB_3246427
Cyclin D1	Cell Signaling Technology	2922	Rabbit	1:1000	AB_2228523
Survivin	Cell Signaling Technology	2808	Rabbit	1:1000	AB_2063948
β-Actin	Santa Cruz Biotechnology	sc-47778	Mouse	1:1000	AB_626632
KPL Anti-Rabbit IgG	Sera Care	474-1506	Goat	1:2500	AB_2721171
KPL Anti-Mouse IgG	Sera Care	474-1806	Goat	1:2500	AB_2307348

**Table 2:** List of primer sequences used for quantitative real-time PCR.

Gene	GenBank Accession No.	Primer Sequence (5′ → 3′)
*KRAS*	XM_038438710.1	Forward: TCTTCAGCGCCAGTCTTAGG Reverse: TGGGGCATGTAGAAGGTAGG
*MYC*	NM_001003246.2	Forward: CACCCATCAGCACAACTACG Reverse: TCTACCACTGTCCAACCTCG
*JAK2*	XM_038655369.1	Forward: TCAGATGTCTGGAGCTTTGG Reverse: TCATCTGTCCTTGCTTGTCG
*STAT3*	XM_038676446.1	Forward: GGCCCAATGGAATCAGCTACAG Reverse: CGAAGAATCAAGCAGTTCCTTC
*CCNE1*	XM_038657660.1	Forward: GTTTGCCATGGTCATTAGGG Reverse: ATGTTGTGTGCGTCTTCAGC
*CCNT2*	XM_038426137.1	Forward: ACAAGAAGCCAGTGGAGACC Reverse: CACTTAACAGCGAGTCCAGC
*CDK9*	XM_038678497.1	Forward: CCTCATCGACAAGTTGCTGG Reverse: ACTCGAACATGGACGTCAGG
*CDK4*	XM_038679138.1	Forward: GTGAAGTGGCATTGCTGAGG Reverse: TTCTCAGGTCTTGGTCCACG
*GAPDH*	NM_001003142.2	Forward: CACAGTCAAGGCTGAGAACG
		Reverse: TACTCAGCACCAGCATCACC

## Data Availability

Data are contained within the article.

## References

[1] Odri G.A., Tchicaya-Bouanga J., Yoon D.J.Y., Modrowski D. (2022). Metastatic Progression of Osteosarcomas: A Review of Current Knowledge of Environmental versus Oncogenic Drivers. Cancers.

[2] Szewczyk M., Lechowski R., Zabielska K. (2015). What do we know about canine osteosarcoma treatment? Review. Vet. Res. Commun..

[3] Schmidt A.F., Nielen M., Klungel O.H., Hoes A.W., de Boer A., Groenwold R.H., Kirpensteijn J., Investigators V.S.S.O. (2013). Prognostic factors of early metastasis and mortality in dogs with appendicular osteosarcoma after receiving surgery: An individual patient data meta-analysis. Prev. Vet. Med..

[4] Prabowo Putro Y.A., Magetsari R., Oksikimbawan Tampubolon Y., Faiz Huwaidi A., Rosfadilla A. (2024). Predictive Factors for Lung Metastasis in High-Grade Osteosarcoma: A 5 Years Experience from Tertiary Referral Hospital. Acta Orthop. Belg..

[5] Lee W., Song G., Bae H. (2025). Review of synergistic anticancer effects of natural compounds combined with conventional therapeutics against chemoresistance and progression of pancreatic cancer. Mol. Cell. Toxicol..

[6] Fenger J.M., London C.A., Kisseberth W.C. (2014). Canine osteosarcoma: A naturally occurring disease to inform pediatric oncology. ILAR J..

[7] Simpson S., Dunning M.D., de Brot S., Grau-Roma L., Mongan N.P., Rutland C.S. (2017). Comparative review of human and canine osteosarcoma: Morphology, epidemiology, prognosis, treatment and genetics. Acta Vet. Scand..

[8] Bernardino P.N., Bersano P.R.O., Lima Neto J.F., Sforcin J.M. (2018). Positive effects of antitumor drugs in combination with propolis on canine osteosarcoma cells (spOS-2) and mesenchymal stem cells. Biomed. Pharmacother..

[9] Kong W.J., Zhang H., Song D.Q., Xue R., Zhao W., Wei J., Wang Y.M., Shan N., Zhou Z.X., Yang P. (2009). Berberine reduces insulin resistance through protein kinase C-dependent up-regulation of insulin receptor expression. Metabolism.

[10] Singh I.P., Mahajan S. (2013). Berberine and its derivatives: A patent review (2009–2012). Expert Opin. Ther. Pat..

[11] Chen W., Miao Y.Q., Fan D.J., Yang S.S., Lin X., Meng L.K., Tang X. (2011). Bioavailability study of berberine and the enhancing effects of TPGS on intestinal absorption in rats. AAPS PharmSciTech.

[12] Battu S.K., Repka M.A., Maddineni S., Chittiboyina A.G., Avery M.A., Majumdar S. (2010). Physicochemical characterization of berberine chloride: A perspective in the development of a solution dosage form for oral delivery. AAPS PharmSciTech.

[13] Xu F., Liu M., Liao Y., Zhou Y., Zhang P., Zeng Y., Liu Z. (2022). Improvement of anticancer effect of berberine by salt formation modifications. Phytomedicine.

[14] Ke X., Zhang R., Li P., Zuo L., Wang M., Yang J., Wang J. (2022). Corrigendum to “Hydrochloride Berberine ameliorates alcohol-induced liver injury by regulating inflammation and lipid metabolism”. Biochem. Biophys. Res. Commun..

[15] Li J., Liu F., Jiang S., Liu J., Chen X., Zhang S., Zhao H. (2018). Berberine hydrochloride inhibits cell proliferation and promotes apoptosis of non-small cell lung cancer via the suppression of the MMP2 and Bcl-2/Bax signaling pathways. Oncol. Lett..

[16] Li H.L., Wu H., Zhang B.B., Shi H.L., Wu X.J. (2016). MAPK pathways are involved in the inhibitory effect of berberine hydrochloride on gastric cancer MGC 803 cell proliferation and IL-8 secretion in vitro and in vivo. Mol. Med. Rep..

[17] Murakami T., Bodor E., Bodor N. (2023). Approaching strategy to increase the oral bioavailability of berberine, a quaternary ammonium isoquinoline alkaloid: Part 2. development of oral dosage formulations. Expert Opin. Drug Metab. Toxicol..

[18] Sammarco A., Beffagna G., Sacchetto R., Vettori A., Bonsembiante F., Scarin G., Gelain M.E., Cavicchioli L., Ferro S., Geroni C. (2023). Antitumor Effect of Berberine Analogs in a Canine Mammary Tumor Cell Line and in Zebrafish Reporters via Wnt/beta-Catenin and Hippo Pathways. Biomedicines.

[19] Withrow S.J., Powers B.E., Straw R.C., Wilkins R.M. (1991). Comparative aspects of osteosarcoma. Dog versus man. Clin. Orthop. Relat. Res..

[20] Gao X., Zhang C., Wang Y., Zhang P., Zhang J., Hong T. (2021). Berberine and Cisplatin Exhibit Synergistic Anticancer Effects on Osteosarcoma MG-63 Cells by Inhibiting the MAPK Pathway. Molecules.

[21] Li C.H., Wu D.F., Ding H., Zhao Y., Zhou K.Y., Xu D.F. (2014). Berberine hydrochloride impact on physiological processes and modulation of twist levels in nasopharyngeal carcinoma CNE-1 cells. Asian Pac. J. Cancer Prev..

[22] Zhang H., Li G., Ni L., Wang R., Peng L., Zang L., Lu C., Wang Z., Liu J. (2025). Berberine hydrochloride inhibits the proliferation and metastasis of p53 mutant gallbladder Cancer cells by regulating the IL6/STAT3 pathway. Sci. Rep..

[23] Cahill J.A., Smith L.A., Gottipati S., Torabi T.S., Graim K. (2024). Bringing the Genomic Revolution to Comparative Oncology: Human and Dog Cancers. Annu. Rev. Biomed. Data Sci..

[24] Zong Y., Li H., Liao P., Chen L., Pan Y., Zheng Y., Zhang C., Liu D., Zheng M., Gao J. (2024). Mitochondrial dysfunction: Mechanisms and advances in therapy. Signal Transduct. Target. Ther..

[25] Jang H., Jo H., Lim W., Park S., Choi I.H. (2025). Salvia microphylla essential oil reduces cell viability in cisplatin-resistant clear cell type ovarian cancer cells caused by mitochondrial dysfunction. Mol. Cell. Toxicol..

[26] Kang Q.C., Liao X.L., Du Z. (2025). GPD1 relieves the proliferation, migration and invasion abilities of oral cancer cells by inhibiting mitochondrial function. Mol. Cell. Toxicol..

[27] Moreau B., Nelson C., Parekh A.B. (2006). Biphasic regulation of mitochondrial Ca^2+^ uptake by cytosolic Ca^2+^ concentration. Curr. Biol..

[28] Carreras-Sureda A., Pihan P., Hetz C. (2018). Calcium signaling at the endoplasmic reticulum: Fine-tuning stress responses. Cell Calcium.

[29] Wacquier B., Combettes L., Dupont G. (2019). Cytoplasmic and Mitochondrial Calcium Signaling: A Two-Way Relationship. Cold Spring Harb. Perspect. Biol..

[30] Li Y., Rasheed M., Liu J., Chen Z., Deng Y. (2024). Deciphering the Molecular Nexus: An In-Depth Review of Mitochondrial Pathways and Their Role in Cell Death Crosstalk. Cells.

[31] Heath-Engel H.M., Shore G.C. (2006). Mitochondrial membrane dynamics, cristae remodelling and apoptosis. Biochim. Biophys. Acta.

[32] Chen Y., Zhao M.M., Li X.Q., Liu Y.Y., Shang Y.H. (2025). Lycopene mitigates DHT-induced apoptosis and oxidative stress in human granulosa cell line KGN by regulating the Nrf2 pathway. Mol. Cell. Toxicol..

[33] Xie J., Xu Y., Huang X., Chen Y., Fu J., Xi M., Wang L. (2015). Berberine-induced apoptosis in human breast cancer cells is mediated by reactive oxygen species generation and mitochondrial-related apoptotic pathway. Tumour Biol..

[34] Jaara H.S., Torres S. (2024). Mitochondrial ROS, a trigger for mitochondrial dysfunction and inflammasome activation and a therapeutic target in liver diseases. Explor. Dig. Dis..

[35] Park J.W., Kim S.M., Lee S.Y., Park S.W., Kim J.K. (2025). Cucurbitacin-I exhibits anticancer activity by inducing apoptosis in SKOV3 human ovarian cancer cells. Mol. Cell Toxicol..

[36] Manning B.D., Cantley L.C. (2007). AKT/PKB signaling: Navigating downstream. Cell.

[37] Fang D., Hawke D., Zheng Y., Xia Y., Meisenhelder J., Nika H., Mills G.B., Kobayashi R., Hunter T., Lu Z. (2007). Phosphorylation of beta-catenin by AKT promotes beta-catenin transcriptional activity. J. Biol. Chem..

[38] Li C.-C., Shibu M.A., Kuo W.-W., Kuo Y.-H., Chao Y.-P., Yao C.-H., Bau D.-T., Lio P.-J., Chiang C.-J., Huang C.-Y.J.M. (2025). Nuclear translocation of β-catenin and migration of arecoline-induced oral cancer cells reduced by Taiwanin E via p-GSK3β downregulation. Mol. Cell. Toxicol..

[39] Cui J., Dean D., Hornicek F.J., Chen Z., Duan Z. (2020). The role of extracelluar matrix in osteosarcoma progression and metastasis. J. Exp. Clin. Cancer Res..

[40] Maybee D.V., Cromwell C.R., Hubbard B.P., Ali M.A.M. (2023). MMP-2 regulates Src activation via repression of the CHK/MATK tumor suppressor in osteosarcoma. Cancer Rep..

[41] Xie K., Guo G.J.M., Toxicology C. (2024). TRIM65 regulates prostate cancer cell growth and stemness through the WNT/β-catenin axis. Mol. Cell. Toxicol..

[42] Chen S., Li L. (2022). Degradation strategy of cyclin D1 in cancer cells and the potential clinical application. Front. Oncol..

[43] Gola C., Giannuzzi D., Rinaldi A., Iussich S., Modesto P., Morello E., Buracco P., Aresu L., De Maria R. (2021). Genomic and Transcriptomic Characterization of Canine Osteosarcoma Cell Lines: A Valuable Resource in Translational Medicine. Front. Vet. Sci..

[44] Gardner H.L., Sivaprakasam K., Briones N., Zismann V., Perdigones N., Drenner K., Facista S., Richholt R., Liang W., Aldrich J. (2019). Canine osteosarcoma genome sequencing identifies recurrent mutations in DMD and the histone methyltransferase gene SETD2. Commun. Biol..

[45] Stein T.J., Holmes K.E., Muthuswamy A., Thompson V., Huelsmeyer M.K. (2011). Characterization of beta-catenin expression in canine osteosarcoma. Vet. Comp. Oncol..

[46] Uva P., Aurisicchio L., Watters J., Loboda A., Kulkarni A., Castle J., Palombo F., Viti V., Mesiti G., Zappulli V. (2009). Comparative expression pathway analysis of human and canine mammary tumors. BMC Genom..

[47] Gola C., Licenziato L., Accornero P., Iussich S., Morello E., Buracco P., Modesto P., Aresu L., De Maria R. (2022). The mitotic regulator polo-like kinase 1 as a potential therapeutic target for c-Myc-overexpressing canine osteosarcomas. Vet. Comp. Oncol..

[48] Liu Y., Liao S., Bennett S., Tang H., Song D., Wood D., Zhan X., Xu J. (2021). STAT3 and its targeting inhibitors in osteosarcoma. Cell Prolif..

[49] Fossey S.L., Liao A.T., McCleese J.K., Bear M.D., Lin J., Li P.K., Kisseberth W.C., London C.A. (2009). Characterization of STAT3 activation and expression in canine and human osteosarcoma. BMC Cancer.

[50] Gorski J.W., Ueland F.R., Kolesar J.M. (2020). CCNE1 Amplification as a Predictive Biomarker of Chemotherapy Resistance in Epithelial Ovarian Cancer. Diagnostics.

[51] Karlsson E.K., Sigurdsson S., Ivansson E., Thomas R., Elvers I., Wright J., Howald C., Tonomura N., Perloski M., Swofford R. (2013). Genome-wide analyses implicate 33 loci in heritable dog osteosarcoma, including regulatory variants near CDKN2A/B. Genome Biol..

[52] Peng J., Zhu Y., Milton J.T., Price D.H. (1998). Identification of multiple cyclin subunits of human P-TEFb. Genes Dev..

[53] Ma H., Seebacher N.A., Hornicek F.J., Duan Z. (2019). Cyclin-dependent kinase 9 (CDK9) is a novel prognostic marker and therapeutic target in osteosarcoma. eBioMedicine.

[54] Day P.J., Cleasby A., Tickle I.J., O’Reilly M., Coyle J.E., Holding F.P., McMenamin R.L., Yon J., Chopra R., Lengauer C. (2009). Crystal structure of human CDK4 in complex with a D-type cyclin. Proc. Natl. Acad. Sci. USA.

[55] Zhou Y., Shen J.K., Yu Z., Hornicek F.J., Kan Q., Duan Z. (2018). Expression and therapeutic implications of cyclin-dependent kinase 4 (CDK4) in osteosarcoma. Biochim. Biophys. Acta Mol. Basis Dis..

[56] Guijarro M.V., Ghivizzani C.S., Gibbs P.C. (2014). Corrigendum: Animal models in osteosarcoma. Front. Genet..

[57] Das S., Idate R., Cronise K.E., Gustafson D.L., Duval D.L. (2019). Identifying Candidate Druggable Targets in Canine Cancer Cell Lines Using Whole-Exome Sequencing. Mol. Cancer Ther..

